# Statistical modeling of annual highest monthly rainfall in Zimbabwe

**DOI:** 10.1038/s41598-022-11839-9

**Published:** 2022-05-11

**Authors:** Keith Musara, Saralees Nadarajah, Martin Wiegand

**Affiliations:** 1 Department of Statistics, University of Zimbabwe, Harare, Zimbabwe; 2 Department of Mathematics, University of Manchester, Manchester, M13 9PL UK; 3 MRC Biostatistics Unit, University of Cambridge, Cambridge, CB2 0SR UK

**Keywords:** Climate sciences, Mathematics and computing

## Abstract

The first statistical analysis of maximum rainfall in Zimbabwe is provided. The data are from 103 stations spread across the different climatic regions of Zimbabwe. More than 90% of the stations had at least 50 years of data. The generalized extreme value distribution was fitted to maximum rainfall by the method of maximum likelihood. Probability plots, quantile plots and Kolmogorov–Smirnov tests showed that the generalized extreme value distribution provided an adequate fit for all stations. The vast majority of stations do not exhibit significant trends in rainfall. Twelve of the stations exhibit negative trends and three of the stations exhibit positive trends in rainfall. Estimates of return levels are given for 2, 5, 10, 20, 50 and 100 years.

## Introduction

Zimbabwe is one of the poorest countries in the world. A global business magazine has ranked Zimbabwe as the second poorest country in the world, see https://bulawayo24.com/index-id-news-sc-national-byo-70943.html Its economy in recent years has been battered by lack of rainfall, drought, sanctions, AIDS pandemic, mass unemployment and hyper inflation. One of the major factors has been the lack of rainfall. Zimbabwe has experienced many periods of droughts. The most recent drought has been in December 2019, which ignited the worst hunger crisis the country has faced in nearly a decade. In November 2019, farmers received only 55% of normal rainfall. Livestock losses reached 2.2 million people in urban areas and 5.5 million in rural ones.

The aim of this paper is to provide the first statistical analysis of annual highest monthly rainfall in Zimbabwe. The following questions and more can then be answered: What are the wettest areas with respect to annual highest monthly rainfall? What are the driest areas with respect to annual highest monthly rainfall? Which areas are most variable with respect to annual highest monthly rainfall? Which areas are least variable with respect to annual highest monthly rainfall? The answers to these questions and more could lead to actions (for example, increased agricultural production in wet areas and planting of crops withstanding droughts in dry areas) which may be of help to improve the economy of Zimbabwe.

To the best knowledge of the authors, there have been no papers on maximum rainfall in Zimbabwe. A related paper on minimum rainfall is due to Chikobvu and Chifurira^1^. Focus on maximum rainfall than minimum rainfall is more meaningful. Minimum rainfall will be mostly zero for a country like Zimbabwe.

However, there have been several papers focusing on rainfall (not maximum rainfall) in specific regions of Zimbabwe. For example, Mooring et al.^2^ examined the effect of rainfall on tick challenge at Kyle-Recreational-Park, Zimbabwe; Gargett et al.^3^ examined the influence of rainfall on Black Eagle breeding in the Matobo Hills, Zimbabwe; Bourgarel et al.^4^ studied the effects of annual rainfall and habitat types on the body mass of impala in the Zambezi Valley; Muchuru et al.^5^ assessed variability of rainfall over the Lake Kariba catchment area in the Zambezi river basin; Sibanda et al.^6^ studied long-term rainfall characteristics in the Mzingwane catchment of south-western Zimbabwe; and so on.

Many papers have been published on extreme rainfall from several other African countries. These papers have been written mostly by scientists from the West, with no collaboration with scientists based in Africa; see, for example, Williams et al.^7–9^, Williams and Kniveton^10^, Pohl et al.^11^, De Paola et al.^12^, Woodhams et al.^13^ and Finney et al.^14^. This adds to the sickening attitude that the West has looked to Africa only for exploitation; not stopping with slave trade, not stopping with colonization, not stopping with stealing of minerals to make among others computers, the West continues scientific exploitation of Africa at an alarming level, see Wiegand et al.^15^. This paper is part of a crusade initiated by the second author to empower Africans to conduct their own research, see http://educateafrica.org/.

There are also hundreds if not thousands of papers published on extreme rainfall from outside of Africa. See, for example, Douka and Karacostas^16^ for Greece, Moccia et al.^17^ for Italy, and Ng et al.^18,19^ for Malaysia. But this paper was motivated by the lack of such papers for Zimbabwe and the lack of such papers written by African scientists.

The contents of the paper are organized into the following sections. “Data” section describes the data. “Method” section describes the method used to analyze the data. “Results and discussion” section presents the results of the method and their discussion. The paper is concluded in “Conclusions” section.

## Data

**Figure 1:**
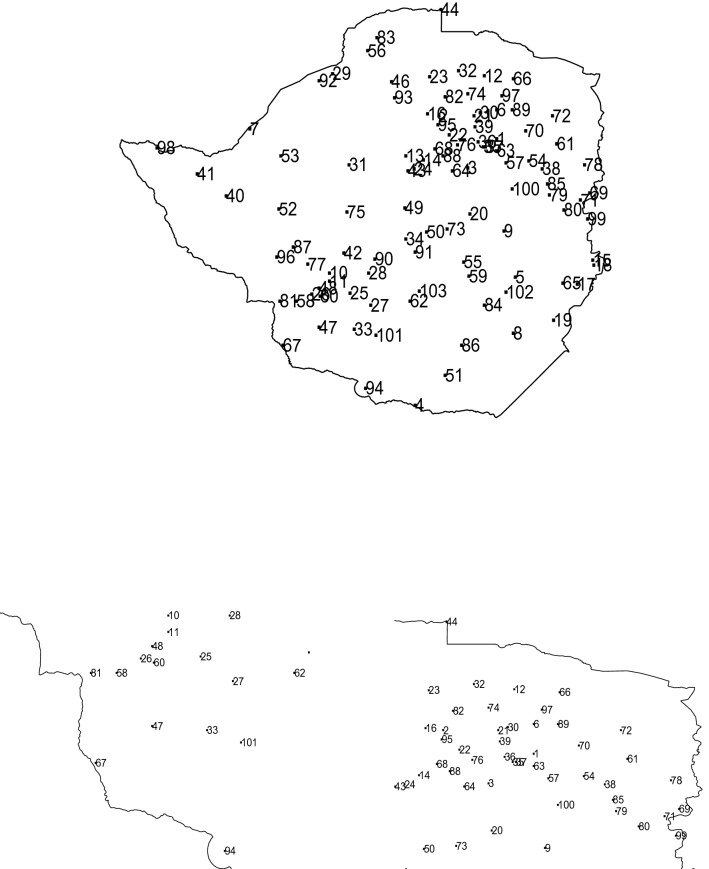
Location of stations as identified by the station numbers in Table 1 (top); Location of stations around Bulawayo, station number 10 (bottom left); Location of stations around Harare, station number 35 (bottom right). ggplot2 version 3.3.5, https://cran.r-project.org/web/packages/ggplot2/index.html was used for plotting.

The data are monthly rainfall in millimetres for 103 stations in Zimbabwe. The station names and years of record are given in Table 1. The locations of the stations are shown in Fig. 1. The stations give a good representation of the geography of Zimbabwe. A large number of stations appears around Harare and Bulawayo, the two largest cities, closeups of the stations around these cities are also shown in Fig. 1. The length of records is reasonable for most stations. The 10th, 25th, 50th, 75th and 90th percentiles of the length are 51, 65, 94, 106 and 114, respectively.

The data were obtained from the Meteorological Services Department, Harare. Many stations for recording rainfall have been discontinued (indicated by $*$ in Table 1) because of the deteriorating economy and lack of resources.

**Table 1:** Station numbers, station names and years of record.

No	Station	Years	No	Station	Years
1	Acturus Mine	1919–2016	53	Lusulu	1949–2016
2	Banket Rail	1909–2016	54	Macheke	1909–2016
3	Beatrice Post Office	1918–2016	55	Makoholi	1919–2016
4	Beitbridge	1921–2016	56	Makuti	1952–2016
5	Bikita Agric	1923–2016	57	Marondera RS Irrig	1940–2016
6	Bindura Rail	1913–2016	58$^{*}$	Marula West	1909–2014
7	Binga	1956–2016	59	Masvingo	1888–2016
8	Buffalo Range	1965–2016	60	Matopos Research Station	1903–2016
9	Buhera	1915–2016	61$^{*}$	Mayo Police	1938–2002
10	Bulawayo Airport	1957–2016	62$^{*}$	Mberengwa DA	1919–2012
11	Bulawayo Goetz	1896–2016	63$^{*}$	Melfort	1947–1970
12	Centenary	1961–2016	64	Mhondoro	1930–2016
13	Dalny Mine	1947–2016	65$^{*}$	Middle Sabi Tanganda	1926–1999
14	Chegutu Rail	1900–2016	66	Mount Darwin	1901–2016
15	Chimanimani DA	1898–2016	67$^{*}$	Mphoengs	1937–1999
16	Chinhoyi	1901–2016	68$^{*}$	Msengezi Experimental Farm	1944–2001
17	Chipinge	1912–2016	69	Mukandi	1918–2016
18	Chisengu	1954–2016	70	Murehwa	1903–2016
19	Chisumbanje	1954–2016	71	Mutare Fire	1899–2016
20	Chivhu	1904–2016	72	Mutoko	1908–2016
21	Concession	1917–2016	73	Mvuma Arex	1911–2016
22$^{*}$	Darwendale Rail	1911–1999	74	Mvurwi	1961–2016
23$^{*}$	Doma Rukute	1964–2001	75	Nkayi	1929–2016
24$^{*}$	Eiffel Flats Blue	1923–2001	76	Norton Rail	1923–2016
25	Esigodini Agric Inst	1942–2016	77$^{*}$	Nyamadhlovu	1906–2009
26$^{*}$	Figtree Police	1911–1999	78	Nyanga Experimental Station	1905–2016
27$^{*}$	Filabusi Police	1902–2003	79	Nyazura Rail	1936–2016
28$^{*}$	Fort Rixon	1904–2003	80	Odzi Police Rail	1911–2016
29$^{*}$	Forthergill	1978–1986	81	Plumtreee	1908–2016
30$^{*}$	Glendale Rail	1917–2011	82$^{*}$	Raffingora Chinomwe	1933–2001
31	Gokwe	1971–2016	83	Rukomechi	1959–2015
32	Guruve	1904–2016	84	Rupike	2015–2016
33$^{*}$	Gwanda Rail	1909–2001	85	Rusape	1904–2016
34	Gweru Thornhill	1898–2016	86$^{*}$	Rutenga	1955–2006
35	Harare Airport	1939–2016	87$^{*}$	Sawmills	1917–2002
36	Harare Belvedere	1891–2016	88	Selous	1971–2016
37	Harare Kutsaga	1953–2016	89	Shamva DA	1911–2016
38$^{*}$	Headlands Rail	1916–2013	90	Shangani Rail	1912–2016
39	Henderson	1921–2016	91	Shurugwi	1909–2016
40	Hwange National Park	1941–2016	92$^{*}$	Tashinga	1967–2012
41$^{*}$	Hwange Rail	1909–2011	93$^{*}$	Rugare Tengwe Thurlaston	1952–2002
42$^{*}$	Inyati	1902–2001	94$^{*}$	Tuli Police	1898–2001
43	Kadoma Cotton Research Inst	1908–2016	95	Trelawney West Enton	1979–2016
44	Kanyemba	1965–2016	96	Tsholotsho	1946–2016
45	Kariba Airport	1962–2016	97$^{*}$	Umpfurudzi	1975–2012
46	Karoi	1925–2016	98	Victoria Falls	1905–2016
47	Kezi	1915–2016	99$^{*}$	Vumba National Park	1929–2005
48$^{*}$	Khami Rail	1912–2001	100	Wedza	1926–2016
49	Kwekwe	1908–2016	101	West Nicholson	1910–2016
50$^{*}$	Lalapanzi Police Guburie	1914–2009	102	Zaka	1923–2016
51$^{*}$	Lions Den	1973–2004	103	Zvishavane	1921–2016
52	Lupane	1938–2016			

The missing values for each station contributed less than 10% of the total period. They were treated as missing in the data analysis reported in “Results and discussion” section. The R software^20^ used for statistical modeling does account for missing values. Data from neighboring stations were compared to see if they were highly inconsistent. Duplication of data values was checked to see if they were real. Values outside of two standard deviations were also checked to see if they were real.

**Figure 2:**
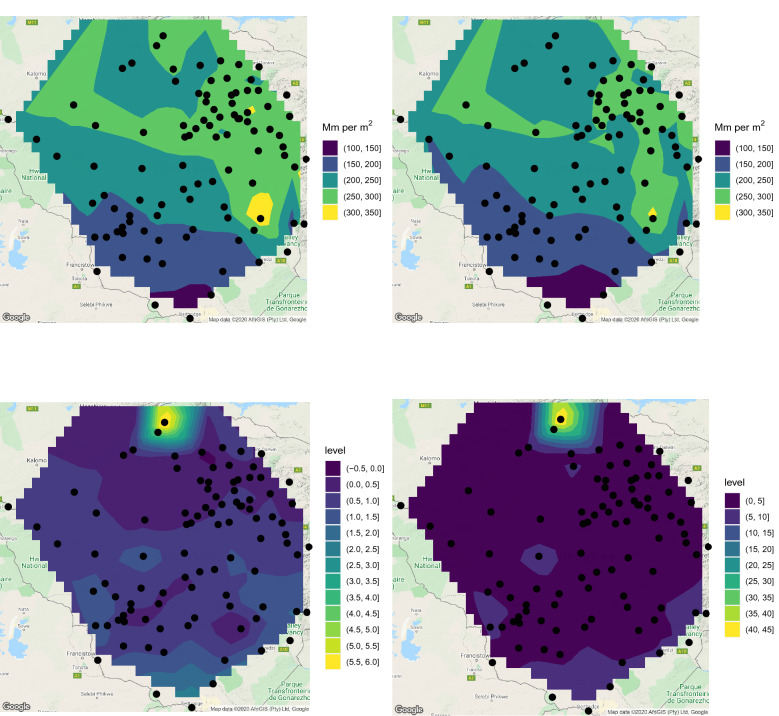
Mean (top left), median (top right), skewness (bottom left) and kurtosis (bottom right) of the annual highest monthly rainfall in Zimbabwe. ggplot2 version 3.3.5, https://cran.r-project.org/web/packages/ggplot2/index.html was used for plotting.

**Figure 3:**
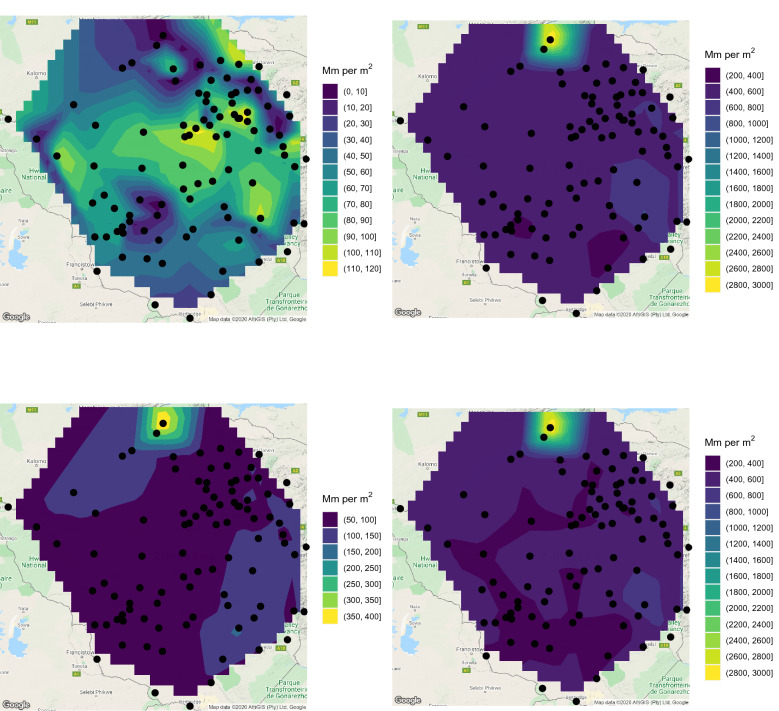
Minimum (top left), maximum (top right), standard deviation (bottom left) and range (bottom right) of the annual highest monthly rainfall in Zimbabwe. ggplot2 version 3.3.5, https://cran.r-project.org/web/packages/ggplot2/index.html was used for plotting.

The annual highest monthly rainfall for each year was recorded as the maximum of the twelve monthly values. Some summary statistics (mean, median, skewness, kurtosis, standard deviation, range, minimum and maximum) of the annual highest monthly rainfall are shown in Figs. 2 and 3. The interpolation used in these two and later figures uses the function interp in the R package interp^20^. The interpolation is based on the algorithms developed by Akima^21,22^ and Renka^23^ which are widely used.

According to mean annual highest monthly rainfall, the wettest areas are those around Harare and that between Masvingo and Mutare. The driest areas are those bordering Botswana and South Africa. The picture is similar for median annual highest monthly rainfall.

Skewness of annual highest monthly rainfall is generally positive. Areas surrounding Chirundu and Kariba give the largest skewness. A small area in the north of the country has negative skewness.

Kurtosis of annual highest monthly rainfall is not far from that of the normal distribution for much of the country. Kurtosis values in areas surrounding Chirundu and Kariba are the largest and correspond to much heavier tails than the normal distribution.

According to standard deviation of annual highest monthly rainfall, the most variable areas are those surrounding Chirundu and Kariba. A big area covering the two major cities, Harare and Bulawayo, shows the least variability. The picture is similar for the range of annual highest monthly rainfall.

According to the minimum of annual highest monthly rainfall, the wettest areas are those close to Chegutu and Harare. The driest areas are those close to the Zambian border and Bulawayo. According to the maximum of annual highest monthly rainfall, the wettest areas are those surrounding Chirundu and Kariba. The driest areas are those surrounding Bulawayo and Renco.

## Method

Let *X* denote a random variable representing the annual highest monthly rainfall. According to extreme value theory (see Leadbetter et al.^24^, Resnick^25^ and Embrechts et al.^26^), the cumulative distribution function of *X* can be approximated by

1 $$\begin{aligned} \displaystyle F_X (x) = \exp \left[ -\left( 1 + \xi \frac{x - \mu }{\sigma } \right) ^{-1 / \xi } \right] \end{aligned}$$

for $\mu - \sigma / \xi \le x < \infty$ if $\xi > 0$, $-\infty< x < \infty$ if $\xi = 0$ and $-\infty < x \le \mu - \sigma / \xi$ if $\xi < 0$, where $-\infty< \mu < \infty$ denotes a location parameter, $\sigma > 0$ denotes a scale parameter and $-\infty< \xi < \infty$ denotes a shape parameter. Note that if $\xi > 0$ then *X* has a heavy tail bounded below by $\mu - \sigma / \xi$. If $\xi < 0$ then *X* has a short tail bounded above by $\mu - \sigma / \xi$.

The distribution in (1) is known as the generalized extreme value (GEV) distribution. The GEV distribution was fitted to the data in “Data” by the method of maximum likelihood, see Coles^27^ for details. The command fgev in the R package evd^20,28^ was used to compute the maximum likelihood estimates. Other distributions (for example, the normal distribution) may provide better fits to the annual highest monthly rainfall. But the GEV distribution is theoretically justified.

Let $\widehat{\mu }$, $\widehat{\sigma }$ and $\widehat{\xi }$ denote the maximum likelihood estimates of $\mu$, $\sigma$ and $\xi$, respectively. A quantity of interest based on (1) is the *T*-year return level loosely interpreted as the annual highest monthly rainfall expected on average once in every *T* years. Let $x_T$ denote the *T*-year return level corresponding to (1). It must satisfy

2 $$\begin{aligned} \displaystyle F_X \left( x_T \right) = 1 - \frac{1}{T}. \end{aligned}$$

Inverting (2),

3 $$\begin{aligned} \displaystyle x_T = \widehat{\mu } + \frac{\widehat{\sigma }}{\widehat{\xi }} \left\{ \left[ -\log \left( 1 - \frac{1}{T} \right) \right] ^{-\widehat{\xi }} - 1 \right\} . \end{aligned}$$

See equation (3.4) in Coles^27^.

### Ethical approval

All authors kept the ‘Ethical Responsibilities of Authors’.

### Consent to participate

All authors gave explicit consent to participate in this study.

### Consent to publish

All authors gave explicit consent to publish this manuscript.

## Results and discussion

The GEV distribution was fitted to the annual highest monthly rainfall data from each of the 103 stations. The estimates $\widehat{\xi }$ were found to be positive for sixteen of the 103 stations. They are Beitbridge, Bikita Agric, Buffalo Range, Buhera, Chisumbanje, Glendale Rail, Kezi, Lupane, Matopos Research Station, Middle Sabi Tanganda, Mphoengs, Nyamadhlovu, Rukomechi, Sawmills, Tashinga and West Nicholson. The distribution of annual highest monthly rainfall for these stations is heavy tailed, meaning that the rainfall recorded at these stations can be unbounded. The distribution of annual highest monthly rainfall for the remaining eighty seven stations is short tailed and is bounded above by $\widehat{\mu } - \widehat{\sigma } / \widehat{\xi }$, which will be referred to as the probable maximum of annual highest monthly rainfall. The estimates of the probable maximum of annual highest monthly rainfall and their standard errors are given in Table 2.

**Table 2:** Estimates and standard errors of probable maximum of annual highest monthly rainfall.

Station	$\widehat{\mu } - \widehat{\sigma } / \widehat{\xi }$ (se)	Station	$\widehat{\mu } - \widehat{\sigma } / \widehat{\xi }$ (se)
Acturus Mine	1464.6 (1352.8)	Lusulu	524.2 (67.7)
Banket Rail	536.1 (62.1)	Macheke	677.9 (137.6)
Beatrice Post Office	1302.4 (1005.5)	Makoholi	8851.0 (68574.1)
Bindura Rail	698.2 (131.2)	Makuti	569.5 (77.9)
Binga	1124.7 (670.4)	Marondera RS Irrig	1307.7 (1217.1)
Bulawayo Airport	958.6 (902.3)	Marula West	1022.9 (484.1)
Bulawayo Goetz	455.4 (66.1)	Masvingo	1291.8 (977.4)
Centenary	2883.0 (8471.1)	Mayo Police	755.1 (223.5)
Dalny Mine	789.8 (342.8)	Mberengwa DA	1691.7 (2912.6)
Chegutu Rail	683.0 (257.9)	Melfort	522.7 (106.8)
Chimanimani DA	8607.2 (30634.7)	Mhondoro	1199.3 (976.8)
Chinhoyi	636.6 (129.0)	Mount Darwin	738.7 (166.2)
Chipinge	2278.6 (2098.6)	Msengezi Experimental Farm	584.5 (90.9)
Chisengu	5534.9 (11950.7)	Mukandi	1112.0 (183.3)
Chivhu	2342.9 (3079.0)	Murehwa	869.4 (172.4)
Concession	1577.9 (1220.4)	Mutare Fire	20114.9 (351565.0)
Darwendale Rail	580.2 (118.6)	Mutoko	787.1 (254.6)
Doma Rukute	471.4 (57.2)	Mvuma Arex	5186.3 (28542.9)
Eiffel Flats Blue	1335.0 (1768.8)	Mvurwi	555.4 (66.8)
Esigodini Agric Inst	664.7 (307.4)	Nkayi	6265.4 (36265.5)
Figtree Police	862.1 (614.6)	Norton Rail	620.6 (148.0)
Filabusi Police	943.0 (454.4)	Nyanga Experimental Station	1071.7 (440.3)
Fort Rixon	657.4 (195.5)	Nyazura Rail	719.7 (199.9)
Forthergill	903.4 (1282.9)	Odzi Police Rail	3767.4 (12013.1)
Gokwe	631.6 (239.9)	Plumtreee	54514.7 (3101996.5)
Guruve	1149.3 (969.1)	Raffingora Chinomwe	472.2 (48.5)
Gwanda Rail	7764.5 (61616.8)	Rupike	301.2 (0.0)
Gweru Thornhill	648.9 (212.8)	Rusape	1701.7 (2160.0)
Harare Airport	1222.7 (1232.8)	Rutenga	1682.2 (2753.8)
Harare Belvedere	6396.7 (37935.7)	Selous	1206.6 (1651.8)
Harare Kutsaga	656.1 (210.4)	Shamva DA	647.7 (70.2)
Headlands Rail	884.8 (271.6)	Shangani Rail	673.5 (136.7)
Henderson	646.5 (89.9)	Shurugwi	1595.7 (1209.4)
Hwange National Park	2319.8 (7006.0)	Rugare Tengwe Thurlaston	657.0 (117.5)
Hwange Rail	1085.9 (705.6)	Tuli Police	1517.8 (2549.1)
Inyati	852.3 (339.6)	Trelawney West Enton	14048.8 (217261.8)
Kadoma Cotton Research Inst	778.8 (298.4)	Tsholotsho	2019.1 (4656.4)
Kanyemba	889.2 (439.6)	Umpfurudzi	651.8 (158.6)
Kariba Airport	501.1 (44.9)	Victoria Falls	1766.1 (2473.5)
Karoi	542.8 (85.1)	Vumba National Park	1471.2 (519.4)
Khami Rail	592.8 (234.1)	Wedza	1406.1 (1231.2)
Kwekwe	1894.5 (2472.2)	Zaka	1379.4 (998.8)
Lalapanzi Police Guburie	1131.2 (645.6)	Zvishavane	845.9 (573.7)
Lions Den	501.8 (76.5)		

The largest of the probable maximum of annual highest monthly rainfall is for Plumtree, and the second largest of the probable maximum of annual highest monthly rainfall is for Mutare Fire, but both have large standard errors. The smallest of the probable maximum of annual highest monthly rainfall is for Rupike. The second smallest of the probable maximum of annual highest monthly rainfall is for Bulawayo Goetz.

In parallel to Table 2, the 100-year return levels of annual highest monthly rainfall for all of the stations were also computed. These estimates and their standard errors are given in Table 3. The largest of the return level is for Rukomechi, and the second largest of the return level is for Chisengu, but one of these has a large standard error. The smallest of the return level is for Rupike. The second smallest of the return level is for Tuli Police.

**Table 3:** Estimates and standard errors of 100-year return level of annual highest monthly rainfall.

Station	Return level (se)	Station	Return level (se)
Acturus Mine	616.2 (358.2)	Lusulu	447.7 (51.2)
Banket Rail	428.3 (38.4)	Macheke	499.7 (73.4)
Beatrice Post Office	526.6 (263.8)	Makoholi	557.3 (2824.2)
Beitbridge	329.6 (157.5)	Makuti	456.5 (52.9)
Bikita Agric	808.5 (630.8)	Marondera RS Irrig	533.2 (305.4)
Bindura Rail	515.7 (68.3)	Marula West	475.7 (149.4)
Binga	583.5 (236.7)	Masvingo	514.2 (264.5)
Buffalo Range	548.4 (587.3)	Matopos Research Station	477.3 (562.4)
Buhera	656.4 (1158.4)	Mayo Police	507.7 (107.1)
Bulawayo Airport	376.7 (220.0)	Mberengwa DA	426.2 (482.0)
Bulawayo Goetz	354.5 (38.5)	Melfort	447.3 (73.3)
Centenary	543.5 (939.1)	Mhondoro	510.6 (260.1)
Dalny Mine	459.3 (128.6)	Middle Sabi Tanganda	423.4 (719.7)
Chegutu Rail	442.2 (107.2)	Mount Darwin	501.6 (77.7)
Chimanimani DA	779.1 (1782.6)	Mphoengs	466.1 (645.2)
Chinhoyi	446.9 (61.4)	Msengezi Experimental Farm	486.4 (64.0)
Chipinge	707.9 (420.3)	Mukandi	865.1 (106.7)
Chisengu	962.7 (1350.2)	Murehwa	561.2 (78.4)
Chisumbanje	515.6 (1506.8)	Mutare Fire	593.6 (6623.9)
Chivhu	556.3 (458.7)	Mutoko	473.4 (100.7)
Concession	613.8 (306.1)	Mvuma Arex	517.6 (1752.6)
Darwendale Rail	414.0 (58.4)	Mvurwi	465.7 (48.4)
Doma Rukute	398.8 (46.9)	Nkayi	491.5 (1744.3)
Eiffel Flats Blue	498.4 (413.5)	Norton Rail	458.1 (78.4)
Esigodini Agric Inst	455.0 (149.5)	Nyamadhlovu	455.6 (548.4)
Figtree Police	413.4 (191.2)	Nyanga Experimental Station	626.2 (168.3)
Filabusi Police	427.2 (138.3)	Nyazura Rail	503.2 (99.9)
Fort Rixon	426.1 (89.1)	Odzi Police Rail	575.7 (1194.0)
Forthergill	523.8 (551.1)	Plumtreee	428.5 (15021.4)
Glendale Rail	590.4 (2649.0)	Raffingora Chinomwe	414.8 (37.2)
Gokwe	467.7 (124.0)	Rukomechi	1020.9 (499.9)
Guruve	463.8 (229.9)	Rupike	300.7 (1.2)
Gwanda Rail	445.2 (2374.6)	Rusape	538.7 (433.7)
Gweru Thornhill	417.9 (90.0)	Rutenga	453.3 (527.8)
Harare Airport	495.3 (308.6)	Sawmills	576.2 (426.8)
Harare Belvedere	546.9 (1907.0)	Selous	500.0 (437.4)
Harare Kutsaga	468.0 (102.4)	Shamva DA	502.1 (45.1)
Headlands Rail	514.3 (110.8)	Shangani Rail	440.4 (64.5)
Henderson	494.0 (52.9)	Shurugwi	709.8 (359.9)
Hwange National Park	460.4 (854.1)	Tashinga	755.8 (1254.0)
Hwange Rail	465.7 (201.1)	Rugare Tengwe Thurlaston	458.5 (62.8)
Inyati	442.0 (116.7)	Tuli Police	323.0 (362.1)
Kadoma Cotton Research Inst	475.7 (116.8)	Trelawney West Enton	601.3 (6042.2)
Kanyemba	496.7 (159.9)	Tsholotsho	419.9 (603.9)
Kariba Airport	417.4 (38.1)	Umpfurudzi	483.3 (94.6)
Karoi	440.4 (51.4)	Victoria Falls	489.1 (416.0)
Kezi	425.6 (7980.3)	Vumba National Park	940.8 (226.8)
Khami Rail	382.8 (101.5)	Wedza	567.1 (323.1)
Kwekwe	518.0 (426.9)	West Nicholson	459.4 (720.5)
Lalapanzi Police Guburie	561.5 (209.6)	Zaka	568.4 (275.6)
Lions Den	430.6 (63.4)	Zvishavane	418.9 (190.5)
Lupane	464.5 (6103.6)		

However, many of the locations in Tables 2 and 3 have large standard errors compared to the estimates of probable maximum/100-year return level. In Table 2, they are Acturus Mine, Centenary, Chimanimani DA, Chisengu, Chivhu, Eiffel Flats Blue, Forthergill, Gwanda Rail, Harare Airport, Harare Belvedere, Hwange National Park, Kwekwe, Makoholi, Mberengwa DA, Mutare Fire, Mvuma Arex, Nkayi, Odzi Police Rail, Plumtreee, Rusape, Rutenga, Selous, Tuli Police, Trelawney West Enton, Tsholotsho and Victoria Falls. In Table 3, they are Buffalo Range, Buhera, Centenary, Chimanimani DA, Chisengu, Chisumbanje, Forthergill, Glendale Rail, Gwanda Rail, Harare Belvedere, Hwange National Park, Kezi, Lupane, Makoholi, Matopos Research Station, Mberengwa DA, Middle Sabi Tanganda, Mphoengs, Mutare Fire, Mvuma Arex, Nkayi, Nyamadhlovu, Odzi Police Rail, Plumtreee, Rutenga, Tashinga, Tuli Police, Trelawney West Enton, Tsholotsho and West Nicholson. The conclusions for these locations should be treated with caution.

**Figure 4:**
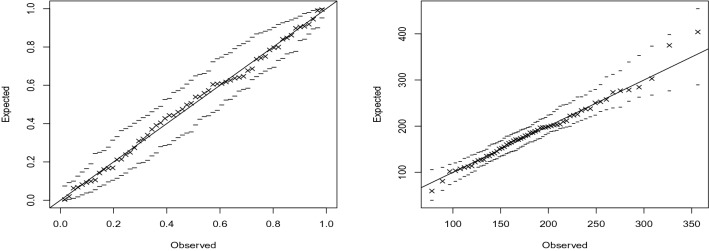
Probability (left) and quantile (right) plots for Bulawayo airport with 95% simulated confidence intervals (dashed lines). R software, version 4.1.2, https://www.r-project.org/ was used for plotting.

**Figure 5:**
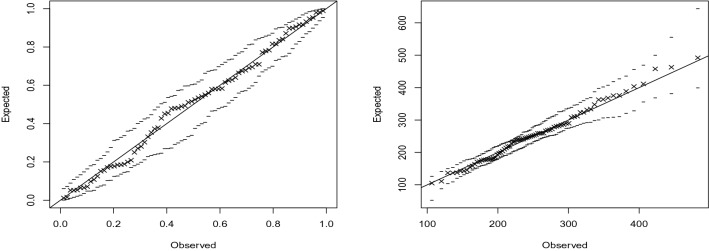
Probability (left) and quantile (right) plots for Harare airport with 95% simulated confidence intervals (dashed lines). R software, version 4.1.2, https://www.r-project.org/ was used for plotting.

The fit of the GEV distribution for each station was checked by probability plots, quantile plots and the Kolmogorov–Smirnov test. The plots are shown in Figs. 4 and 5 for two of the stations. The plots were similar for other stations. The *p*-values of the Kolmogorov–Smirnov test for the two stations were 0.081 and 0.078. The *p*-values for other stations were greater than 0.05 too. Hence, the GEV distribution provides an adequate fit for all stations.

**Figure 6:**
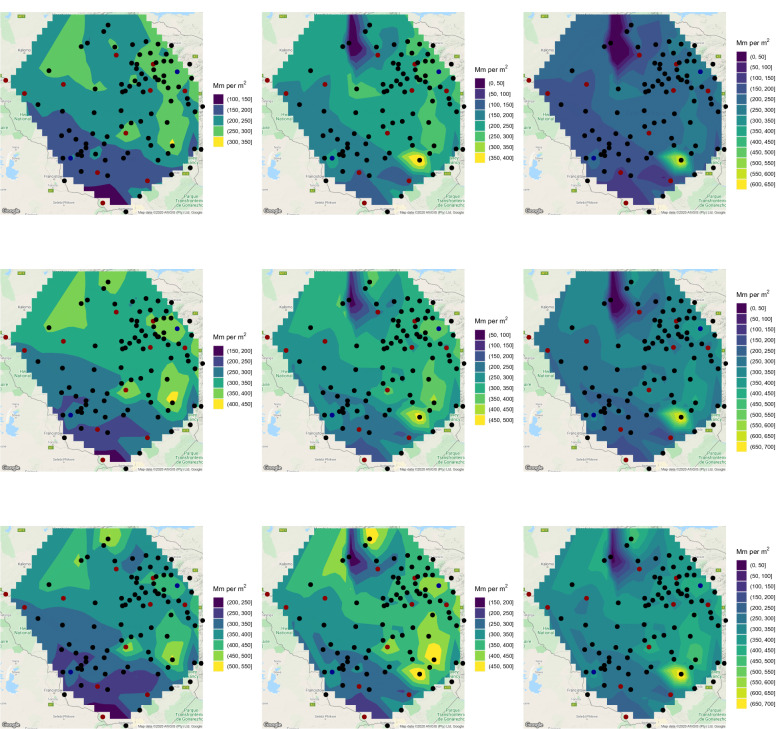
Estimates of 2-year return level (first raw, left), 2-year return level 10 years ahead (first raw, middle), 2-year return level 20 years ahead (first raw, right), 5-year return level (second raw, left), 5-year return level 10 years ahead (second raw, middle), 5-year return level 20 years ahead (second raw, right), 10-year return level (third raw, left), 10-year return level 10 years ahead (third raw, middle) and 10-year return level 20 years ahead (third raw, right). ggplot2 version 3.3.5, https://cran.r-project.org/web/packages/ggplot2/index.html was used for plotting.

**Figure 7:**
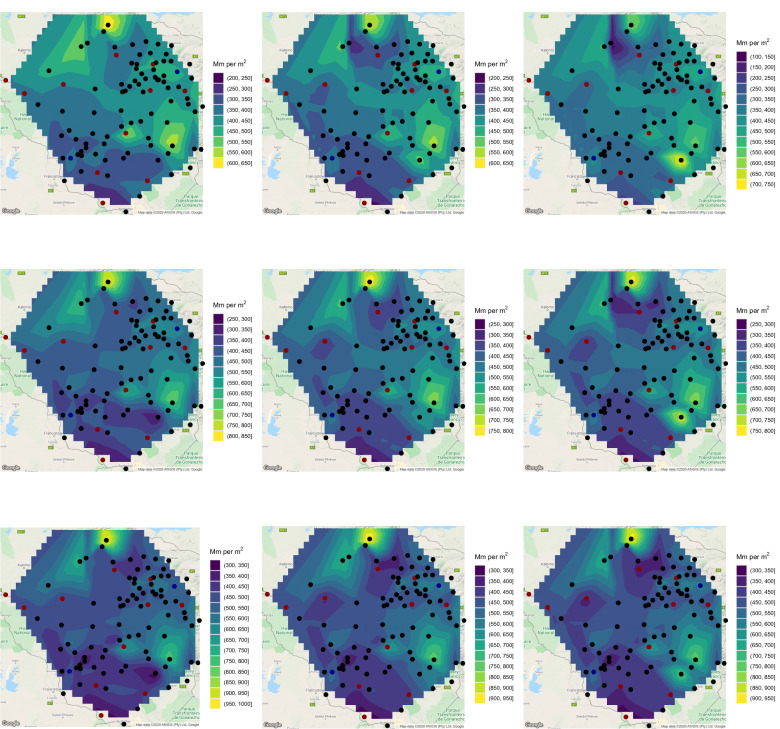
Estimates of 20-year return level (first raw, left), 20-year return level 10 years ahead (first raw, middle), 20-year return level 20 years ahead (first raw, right), 50-year return level (second raw, left), 50-year return level 10 years ahead (second raw, middle), 50-year return level 20 years ahead (second raw, right), 100-year return level (third raw, left), 100-year return level 10 years ahead (third raw, middle) and 100-year return level 20 years ahead (third raw, right). ggplot2 version 3.3.5, https://cran.r-project.org/web/packages/ggplot2/index.html was used for plotting.

Having checked the goodness of fit, (3) was computed for every station and a range of values of *T*. Plots of $x_T$ for $T = 2, 5, 10, 20, 50, 100$ years are shown in Figs. 6 and 7.

According to the 2-year return level, the wettest areas are those around Shurugwi, those around Harare and that between Masvingo and Mutare. The driest areas are those bordering Botswana and South Africa. The picture for the 5-year return level is similar, but the wettest regions are smaller compared to those for the 2-year return level.

According to the 10-year return level, the wettest areas are those around Shurugwi, an area between Masvingo and Mutare and a northern area bordering Zambia. The driest areas are once again those bordering Botswana and South Africa. The picture for the 20-year return level is similar, but the wettest regions are smaller compared to those for the 10-year return level.

According to the 50-year return level, the wettest area is a northern area bordering Zambia. The driest areas are once again those bordering Botswana and South Africa. The picture for the 100-year return level is similar, but the wettest region is smaller compared to that for the 50-year return level.

Finally, significant trends in the annual highest monthly rainfall for each station are investigated. The distribution (1) with the location parameter $\mu = a + b \times \mathrm{Year}$ was fitted, where *b* is the trend parameter. The trend was seen to be significant or not by comparing the fit of this model with the earlier fit of the GEV distribution. Models like $\mu = a + b \times \mathrm{Year} + c \times \mathrm{Year}^2$ and $\mu = \exp \left( a + b \times \mathrm{Year} \right)$ were also fitted, but they did not provide significantly better fits. The methodology used for fitting models like $\mu = a + b \times \mathrm{Year}$ is described in Chapter 6 of Coles^27^.

**Table 4:** Stations exhibiting significant trends in the location parameter.

Station	Trend	$\widehat{a}$	$\widehat{b}$	*p*-value
Beatrice Post Office	Negative	237.7	− 0.598	0.038
Chimanimani DA	Negative	320.1	− 0.733	0.012
Chisengu	Positive	287.0	1.910	0.029
Concession	Negative	298.5	− 1.118	0.000
Gwanda Rail	Negative	167.7	− 0.523	0.040
Headlands Rail	Negative	240.5	− 0.786	0.009
Hwange Rail	Negative	196.9	− 0.474	0.048
Lusulu	Negative	253.8	− 1.323	0.025
Marula West	Positive	127.6	0.792	0.019
Murehwa	Positive	217.4	0.570	0.034
Rutenga	Negative	170.8	− 1.719	0.009
Shurugwi	Negative	307.4	− 0.772	0.031
Rugare Tengwe Thurlaston	Negative	252.8	− 1.834	0.013
Tuli Police	Negative	125.9	− 0.362	0.028
Victoria Falls	Negative	229.2	− 0.379	0.049

Table 4 lists the station names and the parameter estimates of *a* and *b*, and *p*-values showing significance of the trend (since they are all less than 0.05). For the stations not listed in Table 4, the *p*-values were greater than 0.05, hence trends were not significant. Only 15 of the 103 stations exhibit significant trends. Of the 15 stations, 12 stations exhibit negative trends. These stations are plotted in red in Figs. 6 and 7. The remaining 3 stations exhibit positive trends. These stations are plotted in blue in Figs. 6 and 7. The return level estimates 10 years ahead and 20 years ahead of the data records for $T = 2, 5, 10, 20, 50, 100$ years are also shown in Figs. 6 and 7. The return level estimate *m* years ahead of the data records was computed using

$$\begin{aligned} \displaystyle x_T = \widehat{a} + \widehat{b} \left( \text{ Last } \text{ year } \text{ of } \text{ records } + m \right) + \frac{\widehat{\sigma }}{\widehat{\xi }} \left\{ \left[ -\log \left( 1 - \frac{1}{T} \right) \right] ^{-\widehat{\xi }} - 1 \right\} . \end{aligned}$$

The general pattern is that the weather is getting drier with time. However, the changes are statistical significant only at the 15 stations.

The negative trends may be due to climate change or other factors. But this must be treated with caution because seven of the fifteen stations have limited data: Gwanda Rail (1909–2011), Headlands Rail (1916–2013), Hwange Rail (1909–2011), Marula West (1909–2014), Rutenga (1955–2006), Rugare Tengwe Thurlaston (1952–2002) and Tuli Police (1898–2001).

## Conclusions

This paper has provided the first statistical analysis of maximum rainfall in Zimbabwe involving data from 103 stations. The generalized extreme value distribution was shown to provide an adequate fit (as assessed by probability plots, quantile plots and Kolmogorov–Smirnov tests) to data from each station. Eight of the stations (Beatrice Post Office, Chimanimani DA, Concession, Gwanda Rail, Headlands Rail, Hwange Rail, Lusulu, Rutenga, Shurugwi, Rugare Tengwe Thurlaston, Tuli Police and Victoria Falls) exhibit significant negative trends in maximum rainfall. Three of the stations (Chisengu, Marula West and Murehwa) exhibit significant positive trends in maximum rainfall. The remaining stations do not exhibit significant trends.

The wettest areas with respect to 2-year and 5-year return levels are those around Shurugwi, those around Harare and that between Masvingo and Mutare. The wettest areas with respect to 10-year and 20-year return levels are those around Shurugwi, an area between Masvingo and Mutare and a northern area bordering Zambia. The wettest area with respect to 50-year and 100-year return levels is a northern area bordering Zambia. Zimbabwe has taken measures to make good use of the wettest areas. For example, some recent dams built include the Mutange dam built in the Gokwe area in 2016, the Tokwe Mukorsi dam built in the Masvingo area in 2017 and the Kunzvi dam built in the Goromonzi district in 2021.

The driest areas are those bordering Botswana and South Africa. Drought resistent crops (including sunflower, millet, sorghum, bambara nuts and groundnuts) are being grown in these and other areas. Farmers are also using water saving “drip irrigation” methods to grow crops. According to Wikipedia, drip irrigation is a “type of micro-irrigation system that has the potential to save water and nutrients by allowing water to drip slowly to the roots of plants, either from above the soil surface or buried below the surface”.

The results presented in this paper can inform positive actions by the Government of Zimbabwe: for example, further vegetables and other commodities less reliable on rain can be planted on areas showing negative trends; increased agricultural and electricity production based on water can take place in the wettest areas; increased electricity production based on solar energy can take place in the driest areas; and so on.

## Data Availability

The data can be obtained from the corresponding author.

## Code availability

The code can be obtained by contacting the corresponding author.
